# Editorial: Motivations for physical activity, volume V

**DOI:** 10.3389/fpsyg.2026.1947012

**Published:** 2026-08-21

**Authors:** Pedro Morouço, Iuliia Pavlova, Aleksandra M. Rogowska

**Affiliations:** 1 National Program for the Promotion of Physical Activity, Directorate-General of Health, Lisbon, Portugal; 2 Ivan Boberskyj Lviv State University of Physical Culture, Lviv, Ukraine; 3 University of Helsinki, Helsinki, Finland; 4 Institute of Psychology, University of Opole, Opole, Poland

**Keywords:** adaptive systems, behavior change, motivation, physical activity promotion, self-efficacy, socio-ecological model

The public health case for physical activity is no longer in dispute. What remains unresolved is why evidence, opportunity and intention so often fail to become sustained action. The contributions assembled in this Research Topic approach that problem across childhood, adolescence, university life, adulthood, retirement, and clinical care. They examine participation in physical education, organized sport, outdoor recreation, high-intensity interval training, exergaming and rehabilitation, while also considering the social, institutional and technological environments in which movement takes place. Read together, these studies suggest that motivation is neither a stable attribute nor a single psychological quantity. In this Editorial, we interpret motivation as an adaptive system through which people continuously negotiate whether movement is possible, worthwhile, enjoyable, socially supported and compatible with their present lives.

This interpretation matters because conventional physical activity promotion often treats motivation as an input that can simply be increased. Yet a person may value exercise and still lack confidence, opportunity or emotional readiness to act. Conversely, participation initially driven by institutional requirements or social influence may become personally meaningful when it generates competence, enjoyment, identity or connection. The Research Topic therefore moves the field beyond the question of whether motivation matters. It asks how motivational processes are activated, translated into behavior, disrupted by constraints and reorganized as individuals and contexts change.

## Motivation is a pathway, not a starting variable

Several contributions show that motivation is not always associated with physical activity directly. Instead, part of this association may be accounted for by proximal mechanisms that determine whether intention can be enacted. Among Chinese college students, Sheng et al. found that a broad measure of internal exercise motivation was directly associated with physical activity and also indirectly associated through exercise self-efficacy, whereas motivation linked to institutional requirements showed no direct association once self-efficacy was considered. Pan et al. similarly identified exercise self-efficacy as a mediator between physical activity motivation and activity level in medical students, while kinesiophobia weakened the remaining direct association between motivation and activity. Li et al. extended this logic into young adulthood by showing that satisfaction of basic psychological needs in physical education was associated with concurrent exercise behavior through self-efficacy, with gender functioning as a boundary condition. These studies converge on a simple but important proposition: wanting to move and believing one can move are related but non-interchangeable processes.

The same distinction appears in work on more enduring psychological resources. Chen et al. combined variable-centered and person-centered analyses, finding that self-efficacy partly accounted for the association between grit and physical activity, demonstrating the value of studying both average associations and motivational profiles. Outdoor recreation research by Yilmaz likewise positioned self-efficacy as a central resource for campers, illustrating that perceived capability is relevant not only to prescribed exercise but also to voluntary participation in uncertain natural environments. In physically active women, Kabak et al. linked physical activity self-worth with body appreciation through psychological resilience, indicating that positive psychological resources may support a more favorable embodied self-concept rather than merely increasing activity volume.

A second set of studies shows that motivation can also operate through identity and social confidence. Jin et al. reported that exercise identity was associated with college students' perceived social self-efficacy through a chain of mediating processes. This finding shifts attention from “doing exercise” to “being an exerciser”: repeated participation may acquire self-defining meaning, which can then support confidence in social situations. In ultimate frisbee participants, Tian et al. found that leisure motivation was associated with continuous participation intention through leisure involvement and information acquisition. Sustained participation, in this view, develops as individuals invest attention, knowledge and personal significance in an activity.

Taken together, these contributions argue against models in which motivation is treated as a single upstream predictor. Motivation is better understood as a pathway involving perceived competence, efficacy, identity, involvement and interpretation. The practical implication is equally clear. Interventions that attempt to persuade people that exercise is beneficial may fail when they do not also create mastery experiences, realistic progression, meaningful choice and evidence that action is feasible.

## From external conditions to internalized engagement

The Research Topic also demonstrates that motivation is socially produced. Jiang F.-Q. et al. showed that peer support was associated with college students' physical exercise through control beliefs and subjective experience, while intrinsic value moderated several later links in this process. Song and Yang similarly linked students' perceived campus sport environment to physical fitness through peer support and perceived physical literacy. These findings position the social and material environment not as background scenery but as an active component of motivational development. A supportive environment can make activity visible, normal and accessible; it can also provide feedback through which individuals reinterpret their own competence.

At an earlier stage of life, Zheng et al. identified an association between parental support and community-resource use for physical activity with fundamental movement skills in children. This is especially important because motivational adaptation begins before individuals can articulate formal exercise goals. Children who receive support and access to community activity resources are more likely to acquire both the skills and the confidence from which later motivation can develop. Çifçi and Demir broadened this perspective through a socio-ecological analysis of Turkish school-aged adolescents, integrating personal and psychological characteristics with social and environmental factors. Their work reinforces the need to avoid attributing inactivity solely to insufficient willpower when behavior is nested within families, schools, neighborhoods and cultural expectations.

The role of institutional environments is more ambiguous. Formal requirements can initiate participation, but their capacity to sustain it depends on internalization. The contrast observed by Sheng et al. between intrinsic and institutional motivation suggests that compulsory structures are most productive when they build self-efficacy rather than merely enforce attendance. Xiang's study of university students adds an important educational pathway: perceived physical literacy was positively associated with subjective wellbeing both directly and indirectly through autonomous sport motivation. Physical education may therefore become motivationally consequential when it equips students to understand, value and take responsibility for movement, rather than simply requiring temporary compliance.

Social influence also operates through enjoyment and engagement. Kaçay et al. identified enjoyment as a motivational driver of sport continuation, with engagement and social support participating in a moderated mediation process. This work helps explain why technically sound programs can still lose participants: an activity that is beneficial but affectively unrewarding is unlikely to become self-sustaining. The qualitative study by Wendi et al. reached a compatible conclusion in Chinese college students, documenting exercise motives, perceived barriers and preferences regarding high-intensity interval training. Participants do not respond to an intervention format in the abstract; they evaluate whether it is enjoyable, time-efficient, tolerable, socially acceptable and compatible with their goals.

Min's analysis of adult taekwondo provides a particularly useful illustration of motivational adaptation. By emphasizing entertainment motivation in the redefinition of a martial art as modern leisure, the study shows how established activities can remain relevant when their meaning changes for particular participant groups. The question is not whether a practice possesses one authentic motive, but whether it can offer forms of value that fit contemporary adult lives. Motivation is thus not only located within the participant. It is co-designed through the way activities are framed, taught and experienced.

## Motivation under pressure, vulnerability, and transition

Motivational systems become most visible when they are strained. Several contributions examine psychological distress, fear or vulnerability, showing that barriers do not merely reduce the quantity of motivation; they change the conditions under which motivation can become behavior. Ma et al. found that physical activity motivation was inversely associated with anxiety among medical undergraduates through a serial pathway involving smartphone addiction symptoms and sleep quality. The result illustrates how motivation for movement is embedded within a wider behavioral ecology: digital overuse, disrupted sleep and anxiety can form a mutually reinforcing system that competes with active living.

Xu et al. examined physical activity, resilience and depressive symptoms among Chinese college students and identified gender differences in the mediating pathways. Their work underscores that physical activity may be connected to mental health through resources that are distributed and activated differently across groups. Huang et al., studying retired adults, investigated difficulties in emotion regulation and exercise addiction through state anxiety and social dependence. This contribution is a necessary corrective to the assumption that more motivation or more exercise is invariably desirable. Exercise participation can become rigid, compulsive or socially dependent, and healthy participation requires regulation rather than maximum intensity.

Fear-related processes are consequential in both clinical and non-clinical populations. Jiang et al. used the fear-avoidance model to explore experiences associated with kinesiophobia in kidney transplant recipients. Pan et al. likewise showed that kinesiophobia weakened the association between motivation and physical activity among medical students. These studies imply that telling people to overcome fear is inadequate. Fear may reflect bodily uncertainty, prior symptoms, clinical advice, low confidence or catastrophic interpretation. Motivational support must therefore include graded exposure, credible reassurance, symptom interpretation and opportunities to regain trust in the body.

The exploratory secondary analysis by Kuprat et al. extends this concern to people with schizophrenia, examining individual factors associated with adherence to supervised exercise and the relationship between personality characteristics and physical fitness. Clinical exercise programs often demonstrate efficacy among those who attend, while adherence remains the decisive implementation challenge. In this context, motivational adaptation requires attention to affective expectations, previous sport experiences, personality, social support and the practical burden of participation. A program can be clinically appropriate and still be motivationally inaccessible.

Stress also modifies apparently positive motivational processes. Liu D. et al. found that a favorable sport event destination image promoted near-term post-event sport participation through sport attraction, but that perceived stress weakened the translation of attraction into participation. This study is valuable because it captures a common failure point: inspiration generated by an event may be genuine, yet everyday stress can prevent it from becoming action. The legacy of sport events should consequently be evaluated not only by whether spectators develop greater attraction to the sport, but also by whether post-event environments provide low-friction opportunities to participate.

## Inequality, context, and the limits of individual explanation

A major contribution of the Research Topic is its refusal, taken as a whole, to reduce inactivity to individual psychology. Liu W. et al. analyzed social stratification in physical activity participation among Chinese residents through the lenses of spatial and temporal justice. Their study reminds us that people require both places in which to be active and discretionary time in which to use them. Unequal access to safe facilities, transport, leisure time and flexible schedules can produce motivational patterns that are subsequently misread as differences in preference or discipline.

Zhao et al. used interpretable machine learning to examine multilevel predictors of adult physical activity and sport participation during the COVID-19 pandemic. By modeling complex and potentially nonlinear relationships among personal, social and contextual predictors, the study illustrates both the promise and the caution required when applying data-intensive methods to motivation. Machine learning can identify complex patterns and heterogeneous subgroups, but prediction should not be mistaken for explanation. The scientific value lies in combining predictive performance with interpretability and theory, so that identified determinants can inform equitable intervention.

The Research Topic also expands the meaning of participation beyond conventional exercise. Lin et al. examined college students' willingness to engage in sport-based disability assistance volunteering. This work demonstrates that sport-related motivation may include prosocial purpose, inclusion and civic identity. It also challenges a narrow focus on personal health outcomes: participation can be motivated by the opportunity to support others and contribute to a more inclusive sport culture.

The diversity of settings is therefore conceptually important. Motivation in a compulsory physical education class, a transplant rehabilitation pathway, an outdoor camp, an adult taekwondo club, a university sport event or a disability assistance program cannot be assumed to operate identically. What travels across contexts are not fixed motives but recurrent functions: developing competence, experiencing enjoyment, finding meaning, connecting with others, reducing uncertainty and perceiving that participation is realistically possible.

## Digital environments: from novelty to motivational architecture

Digital transformation appears in this Research Topic both as an opportunity and as a source of concern. Kaçay et al. examined AI-related anxiety and employment anxiety among prospective sport managers, highlighting that technological change alters not only service delivery but also professional identity and perceived future security. For the physical activity field, this is a reminder that AI is never motivationally neutral. Systems that promise personalization may simultaneously produce uncertainty, surveillance concerns or diminished autonomy.

Fu et al. developed an evaluation index system for user satisfaction with immersive virtual reality exergaming. Their work is particularly relevant because satisfaction in digital physical activity is multidimensional: technical performance, immersion, usability, safety, enjoyment and perceived value can all determine whether novelty becomes repeated engagement. Digital interventions should therefore be evaluated as motivational environments, not merely as devices. A technically accurate platform that frustrates users, undermines competence or fails to create meaningful progression is unlikely to support sustained activity.

The future lies neither in technological enthusiasm nor rejection. It lies in theory-informed digital design. Wearables, virtual reality, mobile applications and AI can reduce friction, provide timely feedback and adapt challenge to capability. They can also overemphasize metrics, create dependence on external rewards or intensify inequity for people with limited access and digital literacy. The central question is not whether technology motivates, but what form of motivation it cultivates, for whom, under what conditions and for how long.

## Toward adaptive motivational systems

Across the articles in this Research Topic, a coherent agenda emerges. First, future studies should distinguish motivation to initiate activity from the processes required to maintain it. Cross-sectional associations remain useful for theory building, but sustained behavior demands longitudinal, intensive and experimental designs capable of tracking how motives, barriers and contexts change. Second, research should examine transitions. Adolescence, entry into university, retirement, diagnosis, transplantation and the return from a sport event are moments when previous motivational arrangements may no longer fit. These periods offer both heightened risk of disengagement and opportunities for redesign. Third, intervention science should shift from motivational intensity to motivational fit. The goal is not to maximize a generic motivation score. It is to align the form of support with the person's current needs and constraints. Someone with low confidence may require mastery and graded challenge; someone experiencing fear may require safety and symptom interpretation; someone under severe time pressure may require accessible and brief options; someone whose motivation has become compulsive may require flexibility and recovery. Adaptive interventions should therefore respond not only to behavior but to the mechanisms limiting behavior. Fourth, the field should integrate individual and structural explanations. Self-efficacy, enjoyment, identity and autonomous regulation are important, but they operate within distributions of time, space, social support, resources and institutional power. A scientifically mature account of motivation must be able to explain both why two people in similar environments behave differently and why apparently similar motivational interventions produce unequal outcomes across environments. Finally, digital tools should be used to support, rather than replace, human motivational processes. AI-enabled systems could detect changing barriers, tailor feedback and identify periods of vulnerability, but their value will depend on transparency, privacy, inclusivity and the preservation of autonomy. The strongest future models will connect psychological theory, implementation science, socio-ecological analysis, and adaptive technology.

## Conclusion

The contributions to this Research Topic collectively suggest that motivation for physical activity is dynamic, relational and embodied. It is built through experiences of competence, enjoyment, identity and social connection; translated into action through efficacy, opportunity and perceived control; and disrupted by fear, stress, inequality, emotional difficulty and changing life circumstances. Motivation is therefore not a possession that active people have and inactive people lack. It is an adaptive relationship between a person, an activity and a context.

This perspective changes the central scientific question. Rather than asking only how people can be motivated to become active, the field should ask how motivational processes can remain adaptive across the lifespan. Such a shift directs attention toward transitions, feedback loops, implementation and context. It also encourages a more humane practice: instead of attributing disengagement to deficient willpower, researchers and practitioners can identify which component of the person-activity-context relationship no longer fits.

The lasting contribution of this Research Topic is thus not the identification of a universal motive, but the recognition that motivation itself must be adaptive. The future of physical activity promotion will depend not on motivating more people, but on designing systems capable of sustaining long-term engagement as individuals, contexts and societies continue to evolve.

